# Zonisamide for the Treatment of Parkinson Disease: A Current Update

**DOI:** 10.3389/fnins.2020.574652

**Published:** 2020-12-21

**Authors:** Chengqian Li, Li Xue, Yumei Liu, Zhengjie Yang, Song Chi, Anmu Xie

**Affiliations:** ^1^Department of Neurology, Affiliated Hospital of Qingdao University, Qingdao, China; ^2^Department of Medical Record, Affiliated Hospital of Qingdao University, Qingdao, China

**Keywords:** Parkinson’s disease, zonisamide, mechanism, neuroprotection, motor fluctuations, non-motor symptoms, antiparkinsonian drug, pharmacokinetics

## Abstract

Zonisamide has been used as an add-on treatment in order to overcome the deficiencies of the general therapies currently used to resolve the motor complications and non-motor symptoms of Parkinson disease. Various trials have been designed to investigate the mechanism of action and treatment effects of zonisamide in this condition. Most clinical trials of zonisamide in Parkinson disease were from Japan. The vast majority of studies used changes in the Unified Parkinson’s Disease Rating Scale (UPDRS) scores and daily “OFF” time as primary endpoints. Based on adequate randomized controlled trials, zonisamide is considered a safe and efficacious add-on treatment in Parkinson disease. The most convincing proof is available for a dosage of 25–50 mg, which was shown to lead to a significant reduction in the UPDRS III score and daily “OFF” time, without increasing disabling dyskinesia. Furthermore, zonisamide may play a beneficial role in improving non-motor symptoms in PD, including impulsive–compulsive disorder, rapid eye movement sleep behavior disorder, and dementia. Among the various mechanisms reported, inhibition of monoamine oxidase-B, blocking of T-type calcium channels, modulation of the levodopa–dopamine metabolism, modulation of receptor expression, and neuroprotection are the most often cited. The mechanisms underlying neuroprotection, including modulation of dopamine turnover, induction of neurotrophic factor expression, inhibition of oxidative stress and apoptosis, inhibition of neuroinflammation, modulation of synaptic transmission, and modulation of gene expression, have been most extensively studied. This review focuses on structure, pharmacokinetics, mechanisms, therapeutic effectiveness, and safety and tolerability of zonisamide in patients with Parkinson disease.

## Introduction

After Alzheimer disease, Parkinson disease (PD) is the most prevalent neurodegenerative disorder (de Lau and Breteler, 2006; Yang and Perry, 2009). The primary pathological manifestation of PD is the reduction of dopamine-producing cells in the nigrostriatal pathway, which causes a significant reduction in the dopamine levels of the striatal cells (Greenamyre and Hastings, 2004; Liu et al., 2020). However, the etiology of and the mechanisms underlying the decrease in dopaminergic neurons remain unclear. At the moment, dopamine replacement drugs, such as dopamine agonists and levodopa, are generally efficacious in PD patients (Chen and Swope, 2007). Nevertheless, the effectiveness of levodopa and dopamine agonists is gradually lost, and disabling motor complications worsen as time passes, as the current drugs do not slow or halt the progression of neurodegeneration (Bonuccelli and Del Dotto, 2006; Lew, 2007). Non-motor symptoms also appear and threaten patients’ quality of life as the disease progresses (Schapira, 2007). Therefore, there remains a need for new therapeutic strategies (Miwa, 2007). Zonisamide (ZNS) may be an effective therapeutic agent, with varied mechanisms of action relevant to the treatment of PD.

ZNS is a sulfonamide developed in Japan (Uno et al., 1979; Murata, 2004; Bermejo and Anciones, 2009). It has been approved for the treatment of seizures in Japan since 1989 and is commercially available worldwide (Miwa, 2007). Thereafter, ZNS has been authorized as an add-on treatment, along with levodopa, for PD patients in Japan since 2009 (Yang and Perry, 2009). In 2000, Murata et al. (2001) serendipitously discovered that ZNS was efficacious for both epileptic seizures and Parkinsonian symptoms in a single patient. Since then, dozens of preclinical and clinical studies have been performed to clarify the mechanisms and therapeutic effectiveness of ZNS, and the related research has progressed rapidly over the last 10 years. Since the last review was published in 2013 (Grover et al., 2013), there has been a need to summarize the studies reported in the interim. This review focuses on the structure, pharmacokinetics, mechanisms, therapeutic effectiveness, and safety and tolerability of ZNS in patients with PD.

## Structure

**FIGURE 1:**
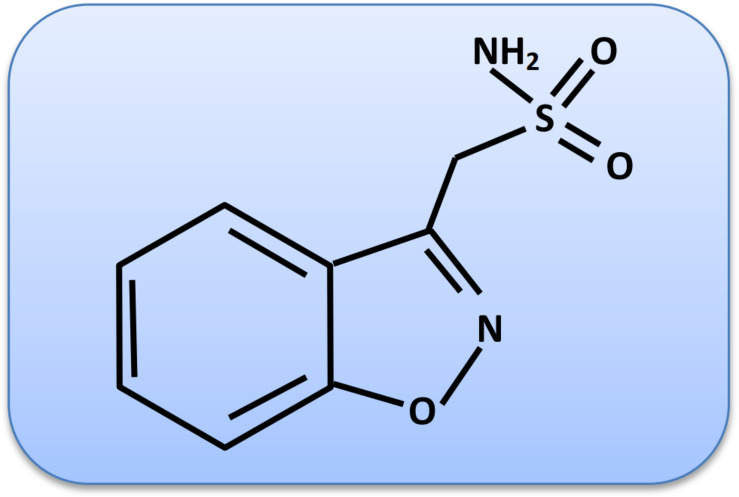
Structure of zonisamide.

ZNS is a sulfonamide drug (Uno et al., 1979). Its chemical name is 1,2-benzisoxazole-3-methanesulfonamide, and molecular weight is 212.227 g/mol. The chemical structure of ZNS is shown in Figure 1.

## Pharmacokinetics

The pharmacokinetics of ZNS has been well investigated and summarized previously (Sills and Brodie, 2007; Yang and Perry, 2009) and are shown in Table 1, based on preclinical or clinical trials, prescribing information, and previous reviews (Ito et al., 1982; Matsumoto et al., 1983; Miwa, 2007; Bermejo and Anciones, 2009).

**TABLE 1:** Properties of zonisamide.

Zonisamide	
Alternative names	AD-810, Zonegran^R^
ATC code	N03A (antiepileptic drugs), N04 (antiparkinsonian drugs)
Mechanisms	See Figure 2
**Prescription**	
Route	Orally
Recommended dosage	25, 50 mg
Administration mode	Once daily
**Indication**	
Recommended as add-on treatment in patients with Parkinson disease	
**Pharmacokinetics** (Uno et al., 1979; Ito et al., 1982; Matsumoto et al., 1983; Stiff et al., 1992; Nakasa et al., 1998; Ohmori et al., 1998; Sills and Brodie, 2007; Yang and Perry, 2009)	
Mean maximum plasma concentration (Cmax)	2.3–12 mg/ml
Median time (tmax)	2.4–3.6 h
Absolute bioavailability	Nearly 100%
Apparent volume of distribution	1.1–1.7 L/kg
Clearance	1.91 L/h
Elimination half-life	49.7–62.5 h
**Pivotal trials**	See Table 2
**Common adverse events**	See Table 3

*ATC, Anatomical Therapeutic Chemical.*

It has been demonstrated that the P450 enzyme CYP3A4 is primarily responsible for the metabolism of ZNS, whereas CYP3A5 and CYP2C19 may also be involved (Nakasa et al., 1998; Ohmori et al., 1998; Morita et al., 2005). All ZNS derivatives, including 2-sulfamoylacetylphenol, N-acetyl ZNS, and unaltered ZNS, are excreted in the urine and feces (Stiff et al., 1992). Notably, potential interactions between ZNS and other medications for PD also need to be taken into account (Sills and Brodie, 2007; Bermejo and Anciones, 2009).

## Mechanisms of Action

The antiparkinsonian mechanism of ZNS is complicated. As the treatment dosage of ZNS in PD is 25–50 mg/day, which is markedly lower than that for the treatment of epilepsy (200–400 mg/day), the mechanism involved is likely to be different (Murata et al., 2007). Here, we will review possible mechanisms, with a particular focus on advances in the last several years, involving both dopaminergic and non-dopaminergic mechanisms.

### Inhibition of Monoamine Oxidase-B

ZNS is capable of inhibiting monoamine oxidase-B (MAO-B) (Uemura et al., 2017). Sonsalla et al. (2010) reported that ZNS regulates MAO-B activity, reversibly, with an IC_50_ of 25 μM *in vitro*.

Previous studies have demonstrated that the metabolism of dopamine by MAO-B produces reactive oxygen species (ROS), which contribute to nigrostriatal degeneration (Alborghetti and Nicoletti, 2019). ZNS prevents the formation of 1-methyl-4-phenylpyridinium [MPP(+)], which is derived from 1-methyl-4-phenyl-1,2,3,6-tetrahydropyridine (MPTP) via MAO-B, and thereby inhibits the oxidation of dopamine to hydrogen peroxide and the related neurotoxic effects (Siebert et al., 2000; Sonsalla et al., 2010).

### Blocking of T-Type Calcium Channels

Previous experimental studies have suggested that patterns of neural firing activity in the basal nuclei in MPTP-induced mice or PD patients are switched to a bursting discharge pattern (Wang et al., 2006; Wichmann and DeLong, 2006; Bermejo and Anciones, 2009). This activity could be reduced by ZNS as this drug blocks the voltage-gated Na^+^ channels and voltage-gated Ca^2+^ channels (T-type calcium channels), resulting in improvement in PD symptoms (Yang et al., 2014; Kunisawa et al., 2018). Additionally, Yurekli et al. (2013) demonstrated that ZNS decreased the level of cytosolic free Ca^2+^ in an MPP-induced neuronal cell model of PD.

### Modulation of Levodopa-Dopamine Metabolism

Nishijima et al. (2018) demonstrated that the effect of ZNS is primarily due to the modulation of levodopa-dopamine metabolism in the striatum. They found that levodopa-induced dyskinesia (LID) was significantly enhanced by prescription of ZNS. On the contrary, apomorphine-induced dyskinesia was not affected by prescription of ZNS. ZNS may enhance dopamine synthesis (Miwa, 2007). ZNS increases the intracellular dopamine concentration when the drug is administered at 25 or 50 mg/kg daily for 3 weeks (Okada et al., 1995). Murata et al. (Murata, 2004) demonstrated that enhanced expression of tyrosine hydroxylase (TH) mRNA contributed to increased dopamine synthesis. They found that levels of TH mRNA were increased in the rat striatum when ZNS was administered at 20 or 50 mg/kg daily for 2 weeks. However, Nishijima et al. (2018) found that a single administration of ZNS did not induce dyskinesia, and TH was not involved in levodopa metabolism. Thus, the enhanced expression of TH cannot fully explain the increased levodopa effect. Further studies are required to demonstrate this mechanism.

It has also been reported that ZNS increases the extracellular dopamine concentration, suggesting that suppression of dopamine reuptake may explain the increased extracellular dopamine concentration (Nishijima et al., 2018). It has been reported that ZNS could increase the release of dopamine (Gluck et al., 2004; Miwa, 2007). However, a recent study showed that ZNS could not elevate striatal expression of vesicular monoamine transferase-2 and dopamine decarboxylase by Western blot analyses, suggesting that it may not be the main mechanism underlying the antiparkinsonian effects of ZNS (Nishijima et al., 2018).

### Modulation of Receptor Expression

Recently, Oki et al. (2017) reported that ZNS could ameliorate LID by modulating the expression of receptors. They designed different models of levodopa-ZNS administration in four groups, namely, intermittent ZNS and levodopa injection, intermittent levodopa injection, continuous levodopa infusion, and no medication. Two weeks after the treatment, they analyzed the mRNA expression of endocannabinoid CB1 receptor, D_1_ and D_2_ receptors, and adenosine A2A receptor in the striatum of PD model rats in each group. Their results indicated that intermittent prescription of levodopa induced LID, which was related to the upregulation of dopamine D_1_ and adenosine A2A receptors. ZNS injection improved LID by downregulation of adenosine A2A and endocannabinoid CB1 receptors.

### Neuroprotection

**FIGURE 2:**
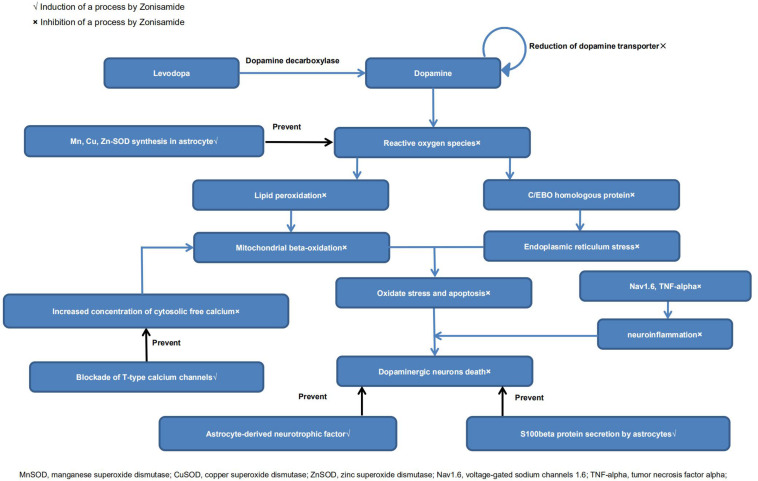
Neuroprotective mechanisms of zonisamide.

It has been reported that ZNS could inhibit the reduction of dopamine-producing cells in neurotoxin-induced animal models of PD (Willmore, 2005; Rosler et al., 2010; Sonsalla et al., 2010; Ikeda et al., 2018). There are indeed a growing number of studies that have explored its neuroprotective effects (Bermejo and Anciones, 2009; Santos, 2012). Below, we review the possible mechanisms. A diagrammatic illustration of the neuroprotective mechanisms of ZNS is shown in Figure 2.

#### Modulation of Dopamine Turnover

Choudhury et al. (2011, 2012) reported that ZNS protected dopamine-producing cells in MPTP-treated models of PD, which may be related to increased S100beta secretion by astrocytes. ZNS also increased dopamine turnover, as surviving dopaminergic neurons recovered the production of dopamine after ZNS prescription (Choudhury et al., 2011). Yokoyama et al. (2010) reported that treatment with ZNS significantly prevented the dopaminergic neuronal damage and proliferation of microglia and increased TH protein levels. Furthermore, they found that ZNS significantly attenuated the motor syndrome a week after MPTP treatment in the behavioral study. Additionally, Ikeda et al. (2018) demonstrated that ZNS treatment delayed the reduction of striatal presynaptic dopamine transporter levels, suggesting that ZNS may play an essential role in slowing the deterioration of early stage PD. They examined the average specific binding ratio (SBR) of dopamine by dopamine transporter single-photon emission computed tomography at the baseline. The SBR decline rate was significantly reduced in the ZNS group, whereas the SBR was significantly reduced in the placebo at the endpoint.

Notably, Yano et al. (2009) mentioned that ZNS could prevent MPTP-induced increases in the levels of glial fibrillary acidic protein in striatal neurons, but Choudhury et al. (2011) came to the opposite conclusion. Further studies are needed to resolve this contradiction.

#### Induction of Neurotrophic Factors

Sano et al. (2015) reported that ZNS could reduce the death of nigrostriatal dopamine-producing neurons via signal transduction by neurotrophic factors. There were increased levels of brain-derived neurotrophic factors in the substantia nigra and striatum in the ZNS group compared with placebo. Choudhury et al. (2012) found that ZNS increased the expression of neurotrophic factors in astrocytes.

An experimental study has reported that ZNS could enhance neurite elongation after nerve injury in primary motor neurons *in vitro* (Yagi et al., 2015). They also found that ZNS could enhance neurite regeneration and protect primary motor neurons against oxidative stress by inducing increased expression of neurotrophic factors (neurotrophin-4/5, brain-derived neurotrophic factors, and nerve growth factors) and their receptors. Further studies are needed to demonstrate whether ZNS has similar effects in nigrostriatal dopaminergic cells.

#### Inhibition of Oxidative Stress and Apoptosis

Some findings have suggested that ZNS exerts neuroprotective effects mainly via antioxidant action. Ueno et al. (2018) reported that long-chain acylcarnitine (LCAC) levels were raised in PD patients after ZNS treatment. As an increase in LCACs implies improvement in mitochondrial beta-oxidation, ZNS may have antioxidant properties. Condello et al. (2013) reported that ZNS could reduce intracellular ROS levels and restore mitochondrial membrane potential. Costa et al. (2010) found that ZNS could protect the striatum against irreversible mitochondrial impairment through a γ-aminobutyric acid (GABA)–mediated mechanism. Choudhury et al. (2012) and Kawajiri et al. (2010) reported that ZNS could increase levels of manganese/copper/zinc superoxide dismutase (Mn/Cu/Zn-SOD) in astrocytes. Yurekli et al. (2013) demonstrated that ZNS could induce modulating effects that increased glutathione (GSH) and glutathione peroxidase levels. Concentrations of cytosolic free Ca^2+^ and lipid peroxidation were also lower in ZNS treatment groups (Yurekli et al., 2013; Ueno et al., 2018). Asanuma et al. (2008, 2010) found that ZNS prevented levodopa-induced dopamine quinone formation in the striatum. They also found that ZNS treatment significantly increased GSH levels by enhancing the expression of cystine/glutamate exchange transporter (xCT). However, Bentea et al. (2017) reached the opposite conclusion. In their experiments, ZNS did not alter GSH levels or influence the expression of xCT both *in vivo* and *in vitro*. More experiments are needed to resolve the contradiction. Moreover, ZNS exerted antiapoptotic effects, as demonstrated by increased cell viability and attenuated caspase-3 activity in human neuroblastoma (SH-SY5Y) cells (Kawajiri et al., 2010; Condello et al., 2013).Tsujii et al. (2015a,b) reported that ZNS had neuroprotective effects against endoplasmic reticulum (ER) stress. ZNS inhibited SH-SY5Y cell death *in vitro* and suppressed ER stress–related cell death *in vivo* via inhibiting the expression of ER stress–induced factors. Furthermore, ZNS suppressed ER stress–related neuron death by inhibiting caspase-3 activation (Yurekli et al., 2013; Tsujii et al., 2015b). Omura et al. (2012, 2013) reported that ZNS inhibited caspase-3 activation via increases in HMG-CoA reductase degradation 1 protein levels. These studies demonstrated that inhibition of ER stress, especially the mechanism via caspase-3, was involved in the neuroprotective actions of ZNS.

#### Inhibition of Neuroinflammation

Hossain et al. (2018) reported that ZNS may potentially modify the disease by inhibition of microglial voltage-gated sodium channels 1.6 (Nav1.6), as well as neuroinflammation. Nav1.6 is expressed in microglial cells of MPTP-induced PD mice and postmortem PD brains, and it contributes to neuroinflammation. ZNS also inhibited the MPTP-induced expression of tumor necrosis factor α and gp91 (phox). Together, these findings suggest that ZNS can reduce neuroinflammation and has neuroprotective potential.

#### Modulation of Synaptic Transmission

It is also possible that ZNS has an effect on synaptic transmission. Experimental studies have suggested that ZNS affects synaptic transmission related to the kynurenine pathway in astroglia (Fukuyama et al., 2014). ZNS could increase the release of metabotropic glutamate-receptor (mGluR) agonists from astrocytes, and activated mGluR agonists inhibited neurotransmission in both direct and indirect pathways, which may explain the efficacy and tolerability of ZNS in PD (Fazio et al., 2012; Copeland et al., 2013; Fukuyama et al., 2014). Yamamura et al. (2009) found that ZNS could influence the level of extracellular neurotransmitters in a dose-dependent manner. Striatal perfusion of ZNS increased the extracellular levels of dopamine in striatal cells and attenuated extracellular levels of GABA in both the subthalamic nucleus and globus pallidus. Extracellular levels of glutamate in the substantia nigra pars reticulata (SNr) were also decreased. Furthermore, they demonstrated that ZNS inhibited the indirect pathways of movement. Sano and Nambu (2019) examined neuronal activities in the SNr and found that ZNS administration showed longer inhibition and reduced late excitation, which suggested that ZNS may enhance the direct pathway, while inhibiting the indirect pathway (Tachibana et al., 2008; Sano et al., 2013).

#### Modulation of Gene Expression

Arawaka et al. (2014) demonstrated that ZNS had neuroprotective effects in a genetic model of PD by attenuating A53T α-synuclein–induced neurotoxicity. They found that ZNS could prevent the loss of dopamine-producing cells by inhibiting a cell death pathway or cellular damage caused by α-synuclein. Furthermore, Ueda et al. (2012) found that ZNS upregulated the expression of several genes associated with nervous system function and development, such as *Gpr143* and *Bdnf*, and of some genes associated with metabolism, including *Aldh1a7*, *Akr1b7*, *Dhdh*, and *Agmat*. Moreover, ZNS downregulated the expression of a few genes associated with inflammation and the immune system.

Recently, Cha et al. (2020) identified a ZNS-responsive gene in PD patients. Carriers of mouse double-minute 4 (*MDM4*) gene had higher ZNS sensitivity, improved motor fluctuation, and reduced “OFF” time. Inhibition of p53 is associated with upregulation of *MDM4*. The association between higher *MDM4* expression and “OFF” time reduction suggested that p53 downregulation may prevent the reduction of dopamine-producing cells and deterioration of PD.

### Other Possible Mechanisms

Recently, some studies have investigated cell replacement therapy using induced pluripotent stem cells (iPSCs) for the treatment of PD (Freed et al., 2001; Olanow et al., 2003; Mendez et al., 2005; Hargus et al., 2010; Rhee et al., 2011). Yoshikawa et al. (2013) reported that ZNS administration improved the survival and differentiation of iPSC-derived dopaminergic neuronal in murine brains. ZNS improved the efficacy of cell replacement therapy for treating PD. Indeed, cell replacement therapy using iPSCs might be a novel therapy strategy for PD in the future.

## Clinical Effectiveness of Zonisamide

Murata et al. (2001) incidentally found that ZNS was effective in treating PD patients. Since then, several clinical trials have been performed to explore the effectiveness of ZNS for the treatment of PD. Based on adequate randomized controlled trials (RCTs), ZNS is efficacious and safe as an adjunctive therapy in patients with PD (Janszky, 2009; Matsunaga et al., 2017). Additionally, the 2018 guideline has supported the use of ZNS for motor symptoms, especially for treating motor fluctuations (Fox et al., 2018). We review the clinical trials below; further details are summarized in Table 2.

**TABLE 2:** Clinical trials on zonisamide effectiveness in the treatment of PD.

Type	Authors (year)	Phase	Length of trial	Enrolled/completed (%)	Inclusion/exclusion criteria	Dosage	Endpoints	Results
Open-label trial	Ikeda et al. (2015)	Phase 2	3 months	10/10 (100%)	H-Y stage I or II Untreated (*De Novo*) patients	25 mg/day for 1 month and 50 mg/day for the next 2 months	UPDRS I-IV Tremor-related UPDRS (items 16, 20, and 21) Sleep condition	I.UPDRS III and tremor-related scores: markedly decreased (*P* < 0.01) II. Sleep state: improved markedly in 3 patients with RBD
Open-label trial	Murata et al. (2001)	Phase 2	12 weeks	9/9 (100%)	Advanced-PD patients	50–200 mg/day	UPDRS I-IV (ON and OFF) H-Y stage (ON and OFF) “OFF” time	I. UPDRS II (OFF): clearly diminished (22.3–12.3, *P* < 0.001) II. H-Y stage (OFF): clearly improved (3.8–2.8, *P* < 0.01) III. “OFF” time: significant decreased (5.9–1.2 h, *P* < 0.001)
Double-blind RCT	Murata et al. (2007)	Phase 2/3b	UP to 16 weeks, including a 2 week run-in period	347/279 (80.4%)	Advanced-PD patients	25, 50, 100 mg/day or placebo	Primary: Variation in UPDRS III Others: Variation in the percentage of patients with ≥ 30% reduction in UPDRS III (responders) Variation in “OFF” time	I. ZNS 25–50 mg: clearly efficacious in diminishing UPDRS III scores II. ZNS 50 mg: percentage of responders was markedly higher III. ZNS 50 mg and 100 mg: clearly efficacious in diminishing “OFF” time
Double-blind RCT	Murata et al. (2015)	Phase 3	16 weeks, including a 4 week run-in period	422/354 (83.9%)	Advanced-PD patients Presence of motor complications (> 2 h “OFF” time/day)	25, 50 mg/day or placebo	Primary: Variation in daily “OFF” time Others: Variation in UPDRS and PDQ-39 Variation in the percentage of patients with ≥ 20% reduction in “OFF” time (responders)	I. ZNS 50 mg: clearly efficacious in diminishing “OFF” time (*P* = 0.005, difference, −0.709 h/day) II. ZNS 50 mg: percentage of responders was markedly higher (40.5%; *P* < 0.001). III. ZNS 50 mg: clearly efficacious in diminishing UPDRS II (off) and UPDRS III
Double-blind RCT	Murata et al. (2016) Registration number: JapicCTI-101198	Phase 3	14 weeks, including a 2 week run-in period	196/166 (84.7%)	Advanced-PD patients Deterioration of response to levodopa	25, 50 mg/day or placebo	Primary: Variation in UPDRS III scores Others: Variation in the percentage of patients with ≥ 30% reduction in UPDRS III (responders) Variation in UPDRS II scores	I. ZNS 25 mg: clearly efficacious in diminishing UPDRS III scores. (*P* = 0.029) II. ZNS 50 mg: percentage of responders was markedly higher (*P* = 0.038) III. ZNS 25 mg: clearly efficacious in diminishing UPDRS II (off) score (*P* = 0.039)

*ZNS, zonisamide; RCT, randomized controlled trials; H-Y stage, Hoehn and Yahr stage; UPDRS, Unified Parkinson’s Disease Rating Scale; PDQ-39, Parkinson’s Disease Questionnaire-39; RBD, rapid eye movement sleep behavior disorder.*

### *De novo*/Early Stage PD

A preliminary, open-label (OL) trial (Ikeda et al., 2015) conducted in Japan suggested that a single administration of ZNS was efficacious in improving motor and sleep dysfunction in treatment-naive patients with early stage PD. Moreover, ZNS was recommended as adjunctive therapy in early stage/stable PD according to 2018 guidelines (Fox et al., 2018).

### Advanced-Stage PD

The largest area of the potential use of ZNS is as adjunctive therapy for motor fluctuations (Fox, 2013). ZNS improved the motor functions and wearing-off phenomenon without worsening dyskinesia in advanced-PD patients (Murata, 2009; Fox et al., 2018). Four clinical trials by published in 2001, 2007, 2015, and 2016 Murata et al. (2001, 2007, 2015, 2016), have examined the efficacy of ZNS in 974 advanced-PD patients.

#### Phase 2 Trials

An OL trial on nine patients with advanced PD showed that ZNS has beneficial effects in improving the primary symptoms of PD, including motor fluctuations (Murata et al., 2001). The Unified Parkinson’s Disease Rating Scale (UPDRS) score, Hoehn and Yahr stage (H-Y stage), and “OFF” time per day were assessed after 12 weeks of ZNS treatment. Both the UPDRS score and H-Y stage (ON/OFF) improved, and unexpectedly, the duration of the “OFF” time markedly improved. These effects continued for more than a year, even for advanced-PD patients. Additionally, ZNS has a long half-life (25 mg: 90 h), and its beneficial effects could be maintained throughout a day with a single dose of the drug per day (Murata et al., 2015).

#### Phase 2b/3 Trials

Murata et al. (2007) conducted an RCT in Japan to assess the efficacy of a daily dose of 25, 50, and 100 mg of ZNS in 347 advanced-PD patients. ZNS 25 and 50 mg resulted in significant decreases in UPDRS III total scores from baseline (ZNS 25 mg, −6.3 ± 0.8; ZNS 50 mg, −5.8 ± 0.8, as compared with −2.0 ± 0.8 in placebo; *P* = 0.001 and *P* = 0.003, respectively; Dunnett test). Likewise, the percentage of responders, defined as patients with a reduction of ≥ 30% in UPDRS part III scores, was significantly higher with ZNS 50 mg (38.8%, *P* = 0.018) than with placebo (22.0%). ZNS 50 mg and ZNS 100 mg groups achieved significant improvements in reducing the total “OFF” time [the mean changes were −1.30 h (*P* = 0.014) and −1.63 h (*P* = 0.013), respectively] compared with the placebo group. Importantly, ON time with annoying dyskinesia did not increase in any ZNS group as compared with placebo; in contrast, a reduction in disabling dyskinesia was observed in the ZNS 50 mg group.

#### Phase 3 Trials

Two phase 3 multicenter RCTs (Murata et al., 2015, 2016) have investigated the efficacy of ZNS among 618 advanced-PD patients: the Murata et al. (2015) and Murata et al. (2016) studies.

The Murata, 2015 study (Murata et al., 2015) examined the effectiveness of ZNS in the reduction of “OFF” time in advanced-PD patients with motor fluctuation as compared with placebo. The primary endpoint was the variation in daily “OFF” time from baseline to the end of a 12-week prescription, according to diarized information at the final assessment. ZNS 50 mg met the primary endpoint and had the most significant efficacy, having a significantly longer decrease in daily “OFF” time at week 12 than placebo (ZNS 50 mg daily “OFF” time decrease: −0.719 ± 0.179 h, compared with placebo: −0.011 ± 0.173 h; *P* = 0.005). Moreover, the percentage of participants with a reduction in “OFF” time ≥ 20% (responders) from the beginning of treatment to week 16 was markedly higher in those receiving ZNS 50 mg than those receiving placebo (40.5% for ZNS 50 mg vs. 20.9% for placebo; *P* < 0.001). ZNS 50 mg also resulted in statistically significant improvements in the UPDRS II (OFF) score (*P* = 0.021) and UPDRS III score at week 12 vs. placebo. However, UPDRS I, UPDRS II (ON), UPDRS IV, and Parkinson’s Disease Questionnaire-39 scores did not show statistically significant differences in improvements between ZNS 50 mg and placebo. Moreover, the increase in the dyskinesia duration at week 12 was not statistically significant in those receiving ZNS 25 mg and 50 mg as compared with placebo (*P* = 0.103 and *P* = 0.235, respectively). This study demonstrated that ZNS 50 mg could significantly reduce the “OFF” time and improve the “OFF” status of activity in advanced-PD patients who exhibited the “wearing-off phenomenon.”

The Murata, 2016 study (Murata et al., 2016) explored the effectiveness and safety of ZNS 25 and 50 mg taken orally once daily, as compared with placebo. The primary endpoint was the variation from the beginning of the treatment to the final assessment in UPDRS part III scores. ZNS 25 mg met the primary endpoint at the final assessment, showing a marked reduction in UPDRS III scores than placebo at week 14 (−5.09 ± 0.9 for ZNS 25 mg vs. −2.9 ± 0.9 for placebo, *P* = 0.029). Likewise, ZNS 50 mg resulted in a significant reduction in UPDRS III total scores at week 12 (−6.1 ± 1.0, *P* = 0.049). The percentage of participants with a reduction in UPDRS III scores ≥ 30% from baseline to week 14 (responders) was markedly higher with ZNS 50 mg than with placebo (45.8% for ZNS 50 mg vs. 27.0% for placebo; *P* < 0.038). Furthermore, the UPDRS II (OFF) scores were significantly improved with ZNS 25 mg than with placebo (*P* = 0.039).

### Late-Stage PD

Patients with late-stage PD (LSPD) refer to those whose H-Y stage ≥ 4 while in the “ON” time (Fabbri et al., 2018). In a phase 2 OL study (Murata et al., 2001) by Murata, 4 of 10 patients fulfilled the LSPD definition. Two patients reached significant improvements in the UPDRS and H-Y stage, whereas the other two did not. In another two phase 3 RCTs (Murata et al., 2007, 2015), further analyses of H-Y stage 4–5 patients were not available.

For safety data of the clinical studies above, see *Safety and Tolerability*.

### Effect on PD-Related Tremors

ZNS may have an effect on intractable tremor (Bermejo and Anciones, 2009; Mochio et al., 2012; Kaplan and Tarsy, 2013). A preliminary OL trial (Nakanishi et al., 2003) performed by Nakanishi et al. reported a potentially valuable role of ZNS in PD patients with residual resting tremor. Seven of nine patients (*P* < 0.0017) had a decrease in the scale of tremor with ZNS administration. Iijima et al. (2011) also reported a case where ZNS was efficacious against re-emergent and residual resting tremor in PD. Both re-emergent and intractable resting tremors markedly decreased with a dosage of 100 mg/day. ZNS was well-tolerated, although mild sleepiness was observed. As previously suggested, ZNS had a beneficial effect on essential tremor (ET) (Ondo, 2006, 2007; Zesiewicz et al., 2007; Bermejo et al., 2008). Another study conducted by Bermejo et al. suggested that ZNS was beneficial to patients with comorbid ET and PD (Bermejo, 2007). In fact, ZNS is effective in controlling symptoms of both disorders. ZNS could thus be a good therapeutic option as adjunctive therapy in PD-related tremor (Freitas and Fox, 2016).

### Effects on Non-motor Symptoms

#### Impulsive–Compulsive Disorders

Impulse control behaviors (ICBs) belong to impulsive–compulsive disorders, which are associated with dopamine replacement therapy in PD (Evans et al., 2006; Raja and Bentivoglio, 2012). Recently, Kon et al. (2018) found that patients who developed ICB at final evaluation were prescribed ZNS earlier and at a higher dosage than other patients, suggesting that ZNS may be associated with ICB. In another study, ZNS increased novelty-seeking behaviors in mice, which were risk factors for ICB (Voon et al., 2011; Uemura et al., 2017). Thus, ZNS might be related to the development of impulsive–compulsive disorders (ICDs) in PD. However, an OL trial (Bermejo et al., 2010) performed by Bermejo et al. suggested that ZNS could play an essential positive role in ICDs. The severity of ICBs was significantly reduced, from −5.8 to −4.8 (mean change). It is important that further RCTs estimate the effectiveness of ZNS on ICDs in patients with PD.

#### Rapid Eye Movement Sleep Behavior Disorder

Rapid eye movement sleep behavior disorder (RBD) is considered one of the most prevailing non-motor symptoms in PD patients (Mollenhauer et al., 2016). It may occur several years before the first symptoms of PD or during the progression of PD (Claassen et al., 2010). Recently, Kataoka and Ueno (2012) reported a case in which treatment with ZNS resolved dream-enacting behaviors and vivid nightmares in an early stage PD patient, suggesting that ZNS might be efficacious for the management of RBD. Further studies are required to estimate the effectiveness of ZNS on RBD in early- and advanced-stage PD patients.

#### Dementia

Dementia is described as an essential complication in PD (Iwaki et al., 2019). Iwaki et al. (2019) and Murata et al. (2007) demonstrated that ZNS may reduce the development of cognitive impairment better than other antiparkinsonian drugs. Although the vast majority of trials were performed in the Japanese population, Tombini et al. (2013) reported the case of a 78 years old Caucasian male patient with PD and dementia, who later developed epilepsy. ZNS significantly improved his seizure and extrapyramidal symptoms without affecting his cognitive status. Further RCTs are needed to evaluate the efficacy of ZNS in preventing cognitive decline among PD patients of different ethnicities (Grover et al., 2013; Tombini et al., 2013).

## Safety and Tolerability

Generally, the therapeutic dose of ZNS for epilepsy (300–600 mg/day) is much higher than that needed for PD (Arzimanoglou and Rahbani, 2006; Ohtahara, 2006). We believe that ZNS is a safe treatment for PD as it has been applied for seizures in Japan for more than 30 years and is well-tolerated.

By now, ZNS has been prescribed to 974 PD patients in four clinical trials by Murata et al. (2001, 2007, 2015, 2016) these studies have suggested that ZNS has high safety without a dose-response relationship for common adverse events. Incidences of adverse events associated with ZNS treatment are summarized in Table 3.

**TABLE 3:** Side effects related to ZNS therapy that appear in more than 3% of participants.

Side effects	Phase 3 trials (Murata et al., 2015)				Phase 3 trials (Murata et al., 2016)			Phase 2b/3 trials (Murata et al., 2007)				
			
	PLC	ZNS 25 mg	ZNS 50 mg	Total	PLC	ZNS 25 mg	ZNS 50 mg	PLC	ZNS 25 mg	ZNS 50 mg	ZNS 100 mg	Total
Total	49.6%	57.7%	60.9%	59.3%	65.1%	55.6%	60.3%	65.1%	70.9%	72.9%	79.5%	/
Constipation	1.5%	1.5%	3.1%	2.3%	**6.3%**	1.6%	1.6%	3.6%	**6.3%**	**8.2%**	4.8%	**6.5%**
Nasopharyngitis	**6.9%**	**7.7%**	3.9%	**5.8%**	**11.1%**	4.8%	3.2%	/	/	/	/	/
Bronchitis	2.3%	1.5%	3.1%	2.3%	/	/	/	/	/	/	/	/
Contusion	3.1%	2.3%	1.6%	1.9%	/	/	/	/	/	/	/	/
Blood LDH increased	3.1%	2.3%	3.1%	2.7%	/	/	/	/	/	/	/	/
Blood urea increased	1.5%	3.1%	0.8%	1.9%	/	/	/	/	/	/	/	/
Decreased appetite	3.1%	4.6%	0.8%	2.7%	**6.3%**	4.8%	1.6%	**14.5%**	**5.1%**	**8.2%**	**16.9%**	**10.1%**
Dyskinesia	**7.6%**	**6.9%**	**7.0%**	**7.0%**	1.6%	**8.2%**	3.2%	/	/	/	/	/
Somnolence	2.3%	3.1%	**6.3%**	4.7%	/	/	/	4.8%	1.3%	**15.3%**	**15.7%**	**10.9%**
Insomnia	3.1%	3.1%	0.0%	1.6%	**7.9%**	4.8%	4.8%	/	/	/	/	/
Apathy	/	/	/	/	/	/	/	**6.0%**	**7.6%**	**7.1%**	**10.8%**	**8.5%**
Dizziness	/	/	/	/	/	/	/	**7.2%**	3.8%	**5.9%**	**7.2%**	**5.7%**
Weight loss	/	/	/	/	**6.3%**	0%	4.8%	4.8%	**7.6%**	3.5%	**9.6%**	**6.9%**
Increased in serum CK	/	/	/	/	**6.3%**	4.8%	1.6%	**8.4%**	**8.9%**	**8.2%**	4.8%	**7.3%**

*Only data for adverse effects with an incidence of > 5% were reported in phase 3 trials (Murata et al., 2016) and phase 2b/3 trials (Murata et al., 2007). Adverse events affecting > 5% are in bold font. ZNS, zonisamide; PLC, placebo; LDH, lactate dehydrogenase; CK, creatinine phosphokinase; /, not reported.*

In a phase 2 trial, the reported side effects were dry mouth (*n* = 1) and the exaggeration of dyskinesia (*n* = 4) (Murata et al., 2001). Notably, doses of ZNS in that study were as high as 50–300 mg/day, and the exaggeration of dyskinesia could disappear with a reduction of levodopa dosage.

In a phase 2b/3 trial, the most frequently reported adverse effects in the total ZNS group, as compared with placebo, were apathy (8.5%), constipation (6.5%), weight loss (6.9%), and somnolence (10.9%) (Murata et al., 2007). Although the incidences of some adverse events for ZNS 50 mg and 100 mg were higher than those for the placebo, there were no statistical differences in the incidences of dyskinesia and hallucination, which are both mostly observed adverse effects of antiparkinsonian drugs, between ZNS groups and placebo, indicating that ZNS is well-tolerated in PD patients at doses of 25–100 mg/day.

In the phase 3 Murata et al. (2015) trial, ZNS 25 mg and 50 mg were well-tolerated in PD patients (Murata et al., 2015). The incidences of total adverse events reported in ZNS 25 mg (57.7%) and 50 mg (60.9%) were not statistically different from those in placebo (49.6%; *P* > 0.05). The incidence of somnolence and that of constipation for ZNS 50 mg were higher than that for placebo (somnolence: 2.3% for placebo, 3.1% for ZNS 25 mg, 6.3% for ZNS 50 mg; constipation: 1.5, 1.5, and 3.1%, respectively). Hallucination and dyskinesia did not occur more frequently for ZNS 25 mg or 50 mg than for placebo.

In the phase 3 Murata et al. (2016) trial, ZNS 25 mg and 50 mg were also well-tolerated in PD patients (Murata et al., 2016). The incidences of adverse events reported in the ZNS 25 mg (55.6%) and 50 mg (60.3%) groups were not statistically different from that in placebo (65.1%; *P* = 0.363 and *P* = 0.713, respectively). The incidence of adverse events that occurred in more than 5% of participants was similar in all three groups. As in the Murata et al. (2015) study, dyskinesia and hallucination did not occur more frequently in ZNS 25 and 50 mg compared with placebo. The incidences of abnormal changes in vital signs, 12-lead resting electrocardiogram, and laboratory tests were low in all groups.

Moreover, a large-scale study in Japan compared ZNS with other antiparkinsonian drugs by analyzing the associations between the administration of eight different varieties of antiparkinsonian drugs and the incidence of PD-relevant symptoms (Iwaki et al., 2019). Iwaki et al. conducted this study based on real-world data from Japan for 2008 to 2014. They demonstrated that ZNS had lower incidences of insomnia, gastric ulcers, and dementia than three of seven other anti-PD drugs (*P* < 0.05).

It is important that medical doctors should remember to ask patients about sulfa-allergies before prescribing ZNS because it is a sulfonamide (Yang and Perry, 2009).

## Discussion

The sulfonamide ZNS exhibits both dopaminergic and non-dopaminergic mechanisms. It is a T-type calcium-channel antagonist and a reversible MAO-B inhibitor. It could modulate levodopa-dopamine metabolism and expression of various receptors. Most importantly, it has the potential for neuroprotection via various mechanisms, such as modulation of dopamine turnover, induction of neurotrophic factors, inhibition of oxidative stress and apoptosis, inhibition of neuroinflammation, modulation of synaptic transmission, and modulation of gene expression. ZNS has a dose-dependent pharmacokinetic profile and has high bioavailability. It interacts with CYP3A4.

The current studies show that ZNS is effective and safe at 25–50 mg/day as an adjunctive therapy in patients with PD. Given the general beneficial effect and safety profile, we recommend initiating this drug at 25 mg/day and titrating it to 50 mg/day when needed. ZNS 50 mg could reduce the “OFF” time, while not increasing troublesome dyskinesia in PD patients with the “wearing-off phenomenon.” ZNS is well-tolerated, with few associated adverse events.

There are still a few limitations in the administration of ZNS. The vast majority of clinical trials of ZNS have been conducted in Japan, and the antiparkinsonian mechanism of ZNS remains incompletely clarified. Further RCTs are needed to estimate the effectiveness of ZNS in PD patients of different ethnicities. Moreover, further in-depth research on the antiparkinsonian mechanism of ZNS is needed.

## Author Contributions

CL carried out the literature retrieval, wrote the manuscript, and made the tables and figures. LX, YL, ZY, and SC critically modified the manuscript. AX funded this project, critically modified the manuscript, and supervised this work. All authors have seen and approved the final version of the manuscript.

## Conflict of Interest

The authors declare that the research was conducted in the absence of any commercial or financial relationships that could be construed as a potential conflict of interest.
